# Sedentary Behavior and Sleep Duration: Depression Risk Factors in Older Adults Pre- vs Post-Pandemic

**DOI:** 10.1093/geroni/igaf122.2267

**Published:** 2025-12-31

**Authors:** Anna Egbert, Isa-Marie Kreuzinger

**Affiliations:** St. Joseph’s University New York, Brooklyn, New York, United States; St. Joseph’s University, Brooklyn, New York, United States

## Abstract

As the older adult population grows, identifying modifiable lifestyle factors that support mental health is increasingly crucial. The COVID-19 pandemic further disrupted their well-being, exacerbating vulnerabilities. Given their high levels of sedentary behavior and poor sleep—both changeable factors—it is essential to reassess their effects on mental health pre- and post-pandemic. This study examined the relationship between self-reported depressive symptoms (Patient Health Questionnaire–PHQ9), sedentary behavior, and sleep duration using National Health and Nutrition Examination Survey (NHANES) data from U.S. adults aged 60–80 years in 2017-2018 (n = 1,442) and August 2021-August 2023 (n = 1,893). The number of older adults showing minimal to severe levels of depressive symptoms remained rather stable from pre- to post-pandemic (Chi2=2.887, p=.577), with moderate to severe cases increasing from 8% to 9%. Hierarchical regression models showed that race/ethnicity–a significant factor before the pandemic–lost significance post-pandemic, while younger age, female gender, lower education, living alone, and lower economic status remained significant across both periods. Importantly, more time spent on sedentary activities (B=.003, p<.001, CI95%=.002, .004) and inadequate sleep (>7h or < 9h; B = 1.439, p<.001, CI95%=1.038, 1.840) were slightly more strongly linked to depressive symptoms post-pandemic. These findings highlight the need to target sedentary behavior and sleep patterns as modifiable risk factors. Promoting healthier habits could help mitigate mental health risks and improve well-being in this growing population.

